# Using Bayesian networks with Tabu-search algorithm to explore risk factors for hyperhomocysteinemia

**DOI:** 10.1038/s41598-023-28123-z

**Published:** 2023-01-28

**Authors:** Wenzhu Song, Zhiqi Qin, Xueli Hu, Huimin Han, Aizhong Li, Xiaoshaung Zhou, Yafeng Li, Rongshan Li

**Affiliations:** 1 School of Public Health, Shanxi Medical University, No.56 Xinjian South Road, Taiyuan, 030001 Shanxi China; 2 Department of Biochemistry and Molecular Biology, School of Basic Medicine, Shanxi Medical University, Taiyuan, 030001 Shanxi China; 3 Department of Nephrology, Shanxi Provincial People’s Hospital (Fifth Hospital) of Shanxi Medical University, Taiyuan, 030012 China; 4 Shanxi Provincial Key Laboratory of Kidney Disease, Taiyuan, 030012 China; 5 Core Laboratory, Shanxi Provincial People’s Hospital (Fifth Hospital) of Shanxi Medical University, Taiyuan, 030012 China; 6 Academy of Microbial Ecology, Shanxi Medical University, Taiyuan, 030012 China

**Keywords:** Epidemiology, Risk factors

## Abstract

Hyperhomocysteinemia (HHcy) is a condition closely associated with cardiovascular and cerebrovascular diseases. Detecting its risk factors and taking some relevant interventions still represent the top priority to lower its prevalence. Yet, in discussing risk factors, Logistic regression model is usually adopted but accompanied by some defects. In this study, a Tabu Search-based BNs was first constructed for HHcy and its risk factors, and the conditional probability between nodes was calculated using Maximum Likelihood Estimation. Besides, we tried to compare its performance with Hill Climbing-based BNs and Logistic regression model in risk factor detection and discuss its prospect in clinical practice. Our study found that Age, sex, α1-microgloblobumin to creatinine ratio, fasting plasma glucose, diet and systolic blood pressure represent direct risk factors for HHcy, and smoking, glycosylated hemoglobin and BMI constitute indirect risk factors for HHcy. Besides, the performance of Tabu Search-based BNs is better than Hill Climbing-based BNs. Accordingly, BNs with Tabu Search algorithm could be a supplement for Logistic regression, allowing for exploring the complex network relationship and the overall linkage between HHcy and its risk factors. Besides, Bayesian reasoning allows for risk prediction of HHcy, which is more reasonable in clinical practice and thus should be promoted.

## Introduction

Homocysteine (Hcy) represents a sulfur-containing amino acid that is an important intermediate produced during the metabolism of methionine and cysteine and is cytotoxic^1,2^. Hyperhomocysteinemia (HHcy) is when a variety of factors contribute to the accumulation of homocysteine, resulting in increased levels of homocysteine in the blood^3,4^. It has been confirmed that elevated Hcy levels can damage the vascular endothelium, leading to the proliferation of smooth muscle cells and atherosclerosis, becoming an independent risk factor for cardiovascular and cerebrovascular disease^5^. It has been shown that lowering homocysteine concentrations by 3 micromol/l from current levels (achievable by increasing folic acid intake) would reduce the risk of ischaemic heart disease by 16% (11% to 20%), deep vein thrombosis by 25% (8% to 38%), and stroke by 24% (15% to 33%)^6^. Also, A 5-mumol/L tHcy increment elevates CAD risk by as much as cholesterol increases of 0.5 mmol/L (20 mg/dL)^7^. A study in the New England Journal of Medicine reported that older adults with high homocysteine in their blood were nearly doubling the likelihood of developing Promencian dementia^8^. Studies at Tohoku University in Japan have also demonstrated that the higher Hcy levels, the more severe the brain damage^9^, the higher the serum Hcy level, the more severe the calcification of intracranial arteries, the heavier the burden of atherosclerosis^10^. Additionally, Hcy is also associated with Alzheimer’s disease^11^, depression^12^, adverse pregnancy outcomes^13^, tumors^14^, and other diseases. It brings serious harm to people's health. A study based on the 2018 Screening Program for High-Risk Groups of Stroke in China found that 181.3 million people have HHcy^15^. The prevalence of HHcy is 39.7% in the overall Chinese population^16^ and 46.23% in older men aged > 60 years^17^. Detecting the risk factors of HHcy and taking some relevant measurements to prevent it is of great significance.

In the past, Logistic regression was employed to explore risk factors for HHcy^18,19^, suggesting that age, BMI, smoking, diet, total cholesterol levels and sex are significantly associated with HHcy. Yet, the model comes with some limitations. The first one concerns independent variables^20^. In clinical research, correlation often exists in influencing factors, and the model is unable to meet the prerequisite of independence between variables. The second one lies in its inability to make a sequential prediction^21^. When one variable is unavailable, the model doesn't work; but data missing is widespread in clinical research. The third one is that the model fails to identify direct or indirect risk factors^22^.

Yet, Bayesian networks (BNs), proposed by Pearl Judea, could address the above concerns. BNs constitute a directed acyclic graph (DAG), reflecting potential relationships among influencing factors, and conditional probability distribution tables (CPT), which demonstrate correlations between variables^23,24^. BNs hold many advantages, one of which relates to its unstrict statistical hypothesis. Moreover, with known nodes, BNs could infer the probability of unknown nodes, flexibly showing the impact of relevant risk factors on HHcy^25^. Also, BNs could graphically elucidate the complex intrinsic network association between one disease and its related factors, and reveal the direct and indirect factors related to the disease through the complex network relationship. As such, BNs could intuitively reveals the network risk mechanism in a complex linkage between one disease and its causes, making up for the drawbacks of the regression model^26^.

Bayesian network learning refers to the obtainment of complete BNs by existing information. The construction consists of parameter learning and structure learning, which could be achieved using constraint-based algorithm and score-based search algorithm. The former one is subject to the sophisticated judgement of node independence. Also, with more nodes, the independence tests between nodes increase exponentially. As for the latter one, new novel approaches, such as Markov Chain Monte Carlo search^27^, multitasking ant colony optimization algorithm^28^, and multi-population harmony search algorithm^29^ have been proposed; yet these new algorithms are usually utilized to discuss high-order SNP interactions. Another excellent algorithm, Tabu-Search, introduced by Professor Fred Glover^30^, is a smart global optimization algorithm. The algorithm is based on human memory simulation and facilitates the global optimum solution obtained by local neighbourhood searching, allowing for the best score-function BN structure. To the best of our knowledge, no scholars have yet sought to employ BNs for risk factor exploration. If applied, it would be clinically significant.

To this end, the present study was the first to construct a BNs with Tabu-search algorithm with significant variables detected by Logistic regression model to discuss the advantages of BNs, and to explore the complicated network relationship between HHcy and its risk factors, thus, elucidating its feasibility in disease risk factor exploration.

## Results

### Baseline characteristics

In abnormal group, men account for 49.1%, and 35.6% of the abnormal ones aged 51 years to 60 years. Over half of them are less-educated, with 34.4% of them with ≤ primary educational background and 50.2% with ≤ middle educational background. Besides, the annual income is not handsome; 41.6% of them are with an income of < 5 k. More detailed descriptions are listed in Table 1.

**Table 1:** Baseline characteristics and clinical parameters between two groups.

Variables	Assignments	Normal Hcy (N = 4110)	HHcy (N = 8175)	*P*
Sex	Male	1195 (29.1%)	4011 (49.1%)	< 0.001
	Female	2915 (70.9%)	4164 (50.9%)	
Age	40 ~	1113 (27.1%)	1585 (19.4%)	< 0.001
	51 ~	1584 (38.5%)	2907 (35.6%)	
	61 ~	1075 (26.2%)	2568 (31.4%)	
	71 ~	338 (8.2%)	1115 (13.6%)	
Education	≤ Primary	1199 (29.2%)	2816 (34.4%)	< 0.001
	≤ Middle	2154 (52.4%)	4101 (50.2%)	
	≤ High	541 (13.2%)	919 (11.2%)	
	≥ Bachelor	216 (5.3%)	339 (4.1%)	
Income	< 5 k	1742 (42.4%)	3399 (41.6%)	< 0.001
	5 k-10 k	897 (21.8%)	2232 (27.3%)	
	10 k-20 k	467 (11.4%)	805 (9.8%)	
	> 20 k	1004 (24.4%)	1739 (21.3%)	
Exercise	No	2420 (58.9%)	4752 (58.1%)	0.436
	Yes	1690 (41.1%)	3423 (41.9%)	
TG	No	3159 (76.9%)	6437 (78.7%)	0.019
	Yes	951 (23.1%)	1738 (21.3%)	
TC	No	3918 (95.3%)	7862 (96.2%)	0.030
	Yes	192 (4.7%)	313 (3.8%)	
LDL	No	3978 (96.8%)	7990 (97.7%)	0.002
	Yes	132 (3.2%)	185 (2.3%)	
HDL	No	3464 (84.3%)	6540 (80%)	< 0.001
	Yes	646 (15.7%)	1635 (20%)	
FPG	Normal	3519 (85.6%)	7382 (90.3%)	< 0.001
	Impaired	305 (7.4%)	413 (5.1%)	
	High	286 (7%)	380 (4.6%)	
GHb	No	3577 (87%)	7407 (90.6%)	< 0.001
	Yes	533 (13%)	768 (9.4%)	
SBP	Low	701 (17.1%)	1130 (13.8%)	< 0.001
	Normal	2016 (49.1%)	3694 (45.2%)	
	High	1393 (33.9%)	3351 (41%)	
DBP	Low	1514 (36.8%)	2868 (35.1%)	< 0.001
	Normal	1670 (40.6%)	3204 (39.2%)	
	High	926 (22.5%)	2103 (25.7%)	
BMI	Underweight	73 (1.8%)	129 (1.6%)	0.022
	Normal	1655 (40.3%)	3203 (39.2%)	
	Overweight	1772 (43.1%)	3455 (42.3%)	
	Obesity	610 (14.8%)	1388 (17%)	
Smoking	No	3454 (84%)	5905 (72.2%)	< 0.001
	Yes	656 (16%)	2270 (27.8%)	
Alcohol	Seldom	3680 (89.5%)	6729 (82.3%)	< 0.001
	Sometimes	379 (9.2%)	1237 (15.1%)	
	Always	51 (1.2%)	209 (2.6%)	
Salt consumption	Light	972 (23.6%)	2263 (27.7%)	< 0.001
	Balanced	2686 (65.4%)	4751 (58.1%)	
	Salt	452 (11%)	1161 (14.2%)	
Diet	Vegetable	1105 (26.9%)	3011 (36.8%)	< 0.001
	Balanced	2832 (68.9%)	4770 (58.3%)	
	Meat	173 (4.2%)	394 (4.8%)	
ACR	Normal	3600 (87.6%)	7117 (87.1%)	0.420
	Abnormal	510 (12.4%)	1058 (12.9%)	
MCR	Normal	3710 (90.3%)	7129 (87.2%)	< 0.001
	Abnormal	400 (9.7%)	1046 (12.8%)	

### Univariate analysis

We used chi-square tests to explore the differences in each variable between the normal Hcy group and abnormal Hcy group. The results showed that the differences in exercise and ACR between the two groups were not statistically significant (*P* > 0.05). Sex, age, education, income, triglyceride (TG), total cholesterol (TC), low-density lipoprotein (LDL), high-density lipoprotein (HDL), fasting plasma glucose (FPG), glycosylated hemoglobin (GHb), systolic pressure (SBP), diastolic blood pressure (DBP), body mass index (BMI), smoking, alcohol, diet, MCR were statistically significant between the two groups ( *P* < 0.05), as shown in Table 1.

### Multivariate analysis

We conducted a multivariate logistic regression model with a stepwise method (α_in_ = 0.05, α_out_ = 0.10) for risk factors for HHcy, with HHcy presence as the dependent variable; independent variables were those significantly associated with stroke presence in univariate analysis. The multivariate analysis suggested that HHcy was significantly associated with sex (OR 0.460, CI 0.414, 0.510), age (OR 1.267, CI 1.213, 1.324), FPG (OR 0.804 CI 0.738, 0.875), GHB (OR 0.707, CI 0.615, 0.811), SBP (OR 1.156, CI 1.091, 1.226), BMI (OR 1.098, CI 1.041, 1.158), smoking (OR 1.189, CI 1.050, 1.346), diet (OR 0.674, CI 0.627, 0.725), MCR (OR 1.179, CI 1.034, 1.345), as shown in Table 2.

**Table 2:** Risk factors for HHcy using Logistic Regression.

Variables	B	S.E	*P*	OR (95% C.I.)
Sex	− 0.777	0.053	< 0.001	0.460 (0.414, 0.510)
Age	0.237	0.022	< 0.001	1.267 (1.213, 1.324)
FPG	− 0.218	0.043	< 0.001	0.804 (0.738, 0.875)
GHB	− 0.347	0.070	< 0.001	0.707 (0.615, 0.811)
SBP	0.145	0.030	< 0.001	1.156 (1.091, 1.226)
BMI	0.093	0.027	0.001	1.098 (1.041, 1.158)
Smoking	0.173	0.063	0.006	1.189 (1.050, 1.346)
Diet	− 0.395	0.037	< 0.001	0.674 (0.627, 0.725)
MCR	0.165	0.067	0.014	1.179 (1.034, 1.345)
Constant	0.812	0.116	< 0.001	2.253

### Bayesian networks

**Figure 1:**
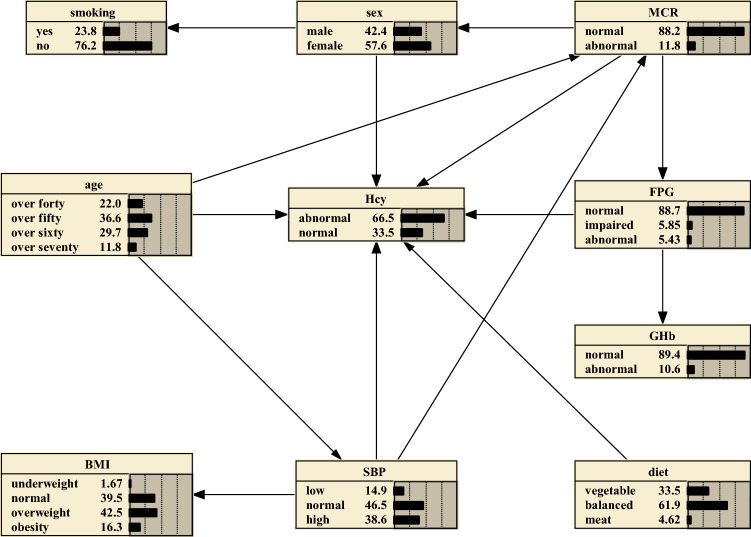
Tabu-search algorithm to construct HHcy Bayesian networks and prior probability. The figure was plotted using Netica (www.norsys.com).

As shown in Fig. 1, BNs were built with 10 nodes and 14 directed edges, which is graphical in demonstrating the risk factors for HHcy. The results suggest that age, sex, MCR, FPG, meat and SBP constitute direct risk factors for abnormal Hcy, and smoking, GHb and BMI represent indirect risk factors for abnormal Hcy. Of note, age could directly influence Hcy, and could indirectly influence it through SBP, showing that SBP could serve as an intermediate link that affects Hcy. FPG could also work as an intermediate link for MCR affecting Hcy.

### Bayesian reasoning

Prior probabilities of the variables are presented in Fig. 1. The resulting probabilistic model could quantitatively analyse the influence of these factors on HHcy via computing conditional probabilities P(y|xi). From Fig. 1, we could learn that the prior probability of HHcy stands at 0.665. If one is subject to high SBP, the probability increases from the prior probability to P (HHcy|high SBP) = 0.701 (Supplementary Fig. 1). And if the person’s age is over seventy, the probability rises to P (HHcy|high SBP, 71–91 years) = 0.746 (Supplementary Fig. 2). If the person has abnormal MCR, the probability rises to P (HHcy|high SBP, 71–91 years, abnormal MCR) = 0.779 (Supplementary Fig. 3).

### Comparison of Logistic regression and Bayesian Networks with two different algorithms

As demonstrated in Table 2, Logistic regression model could only show us the risk factors for HHcy, failing to construct a complicated network relationship between risk factors and HHcy. Besides, it doesn’t allow for Bayesian reasoning. As such, Logistic regression model is relatively less welcome in risk factor detection.

**Figure 2:**
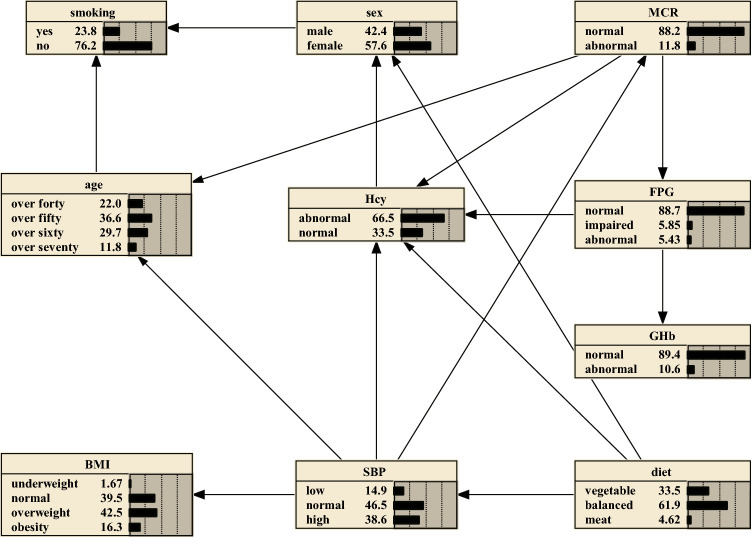
Hill-climbing algorithm to construct HHcy Bayesian networks and prior probability. The figure was plotted using Netica (www.norsys.com).

To further reveal the good performance of Tabu-search algorithm, BNs constructed with Hill-climbing algorithm was also conducted. As shown in Fig. 2, BNs were constructed with 10 nodes and 15 directed edges. Different from Tabu-search algorithm-based BNs, it suggested that age and sex were not direct risk factors for abnormal Hcy, which is clinically irrelevant, because with age increasing, the prevalence of HHcy is obviously higher. Besides, it showed that MCR and SBP are parental nodes for age, namely, they are direct risk factors for age, which is less convincing. Actually, as per the medical knowledge, age should be the parental node for both MCR and SBP which reflect kidney injury and blood pressure, respectively. Overall, BNs constructed with Hill-climbing algorithm is less excellent in exploring the direct edges than that with Tabu-search algorithm.

**Figure 3:**
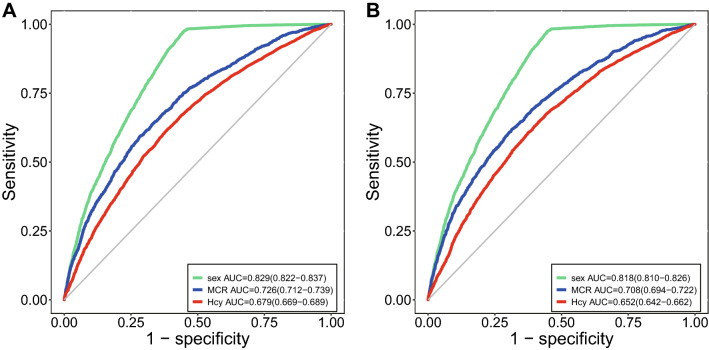
Performance of the Tabu-search algorithm (A) and Hill-climbing algorithm (B) in discussing risk factors for HHcy. The figures were plotted using R software (https://www.r-project.org/).

Besides, receiver operating characteristic (ROC) curve was used to evaluate the performance of the BNs with two algorithms. In Tabu-search algorithm-based BNs, the area under curve (AUC) was 0.829 (95% Confidence Interval (CI): 0.822–0.837) and 0.726 (95% CI 0.712–0.739) for sex and MCR, which are two clinically important indicators for HHcy. Also, the AUC for Hcy was 0.679 (95% CI 0.669–0.689). Yet, in Hill-climbing algorithm, the AUC were 0.818 (95% CI 0.810–0.826), 0.708 (95% CI 0.694–0.722) and 0.652 (95% CI 0.642–0.662) for sex, MCR and Hcy, respectively (Fig. 3). The AUC results suggested that Tabu-search algorithm-based BNs is comparatively better than Hill climbing algorithm-based BNs.

## Discussion

In exploring risk factors for HHcy, Logistic regression was previously employed, which uses probabilities to reflect the associations, but the model fails to elucidate the overall linkage between risk factors and could not offer direct or indirect risk factors. BNs boast advantages over detecting risk factors, the first one relates to its unstrict prior assumptions. Second, the model can integrate different variables and present their relative importance^31^. Accordingly, BNs have been favoured by many clinical researchers in recent years. Studies have shown that BNs, as a risk assessment tool for large clinical datasets, could quantitatively identify important parameters for predicting specific histopathological diagnoses and prognoses^32^, and determining risk factors for diseases that support medical decision-making^33^.

As far as we know, this study represents the first one to employ BNs to detect the risk factors of HHcy. BNs outperforms Logistic regression in risk factor detection. Firstly, combined with previous study using Logistic regression for factors associated with hyperhomocysteinemia^18^ and our comparison between the two models in the Result, BNs facilitates new ideas for disease relationship study and allows for the influencing factors of a certain intermediate link in the occurrence and development of HHcy. Secondly, BNs could offer more valuable information, as it’s represented by “population clustering” we mentioned in the result. The third one relates to interaction. The regression model could only offer those risk factors associated with HHcy, whereas, BNs could facilitate a more in-depth understanding of how they are related to each other and affect the incidence of HHcy with a graphical approach. Also, additional interaction analysis together with Logistic regression is required when interaction exists between variables. However, BNs could allow for direct interaction exploration, offering significant clues for a deeper comprehension of the internal complicated variable relationships.

A cross-sectional study conducted in Shanghai, China suggested that the prevalence of H-type hypertension in patients with primary hypertension was 80.0%^34^. Yet, many individuals show no awareness of HHcy and are inclined to drug administration with ordinary hypertension, with poor treatment effects and unstable blood pressure fluctuations, posing severe damage to blood vessels and the brain. It has been documented that Individuals with H-type hypertension are 12.7 times higher than healthy people to be subject to stroke death^35^. As such, when abnormal blood pressure was detected, individuals are suggested to have their homocysteine tested, and some corresponding measures should be taken to lower its levels. Insulin resistance in diabetics can cause increased blood Hcy^36^. The combination of HHcy with FPG will greatly accelerate the speed of blood vessel lesions, thereby making cardiovascular and cerebrovascular disease worse, and greatly increasing the risk of cardiovascular and cerebrovascular diseases.

MCR represents one of the parameters for tubular injury. Undoubtedly, renal tubules could reabsorb most of the water, glucose, amino acids, sodium, phosphate, etc. in the original urine, they also could secrete or excrete substances produced by themselves or in the blood into the urine, such as H^+^, creatinine and certain drugs, and regulate water, electrolytes and acid–base balance. Normally, Hcy could be decomposed through two metabolic pathways, one of which concerns kidney excretion^37^ and under normal circumstances, Hcy can be decomposed in the body and excreted out of the body through the kidneys, however, when there is a problem with the renal tubules, the excretion capacity of the kidneys will also decline, and the homocysteine in our human body will not be able to be discharged smoothly, and the long-time accumulation of Hcy would threaten blood vessels, which in turn may lead to the occurrence of HHcy^38^. The further accumulation of Hcy will further lead to the formation of CKD, thus developing a vicious cycle and posing a huge threat to human health. It has been shown that HHcy occurs in approximately 85% of CKD patients with impaired renal metabolism and reduced renal excretion^39^. Elevated Hcy levels are an independent predictor of rapid renal decline and CKD events in hypertensive populations in China^40^.

Amid the ageing population in China, the prevalence of hypertension, diabetes mellitus and other chronic diseases has risen sharply, especially in rural areas of China subjected to relatively backward medical conditions and less access to regular medical examination accompanied by poor health awareness^41,42^. The elderly often comes with HHcy and other several conditions, exacerbating the possibility of cardiovascular and cerebrovascular development, becoming the “silent killer” of health^43^. It’s encouraged to take regular Hcy monitoring and lifestyle adjustment, including eating more fresh fruit, supplementing microbial B and folic acid, reducing tobacco intake, and participating in sports exercises, including jogging, cycling, and Tai Chi.

This study should be interpreted in the context of several limitations, one of which concerns the BNs model itself. Since the model is data-driven, it could not reflect a causal relationship, but a correlation between variables. Second, in this cross-sectional study, some data were collected using questionnaires and some variables were not well-defined, such as smoking and alcohol consumption, and some results may be subject to recall bias. Third, the study subjects were recruited from ten rural areas in Shanxi Province aged over 40 years old, and more external data should be included in the modeling. Our ongoing work will focus on data from other provinces, as well as other age groups to build a more accurate model. Last, The BN model obtained in this study is based on Tabu-Search algorithm, which may not be the best. In our future work, we would try to employ novel algorithms to construct a better BN model.

In conclusion, our study demonstrates that BNs combined with Tabu-search algorithms represent a good approach to detecting risk factors for HHcy. It could offer a scientific idea for clinical practice and should be applied in medical research. Besides, regular monitoring of homocysteine levels is essential for individuals to lower its occurrence and to prevent the prevalence of its related diseases.

## Materials and methods

### Study participants

**Figure 4:**
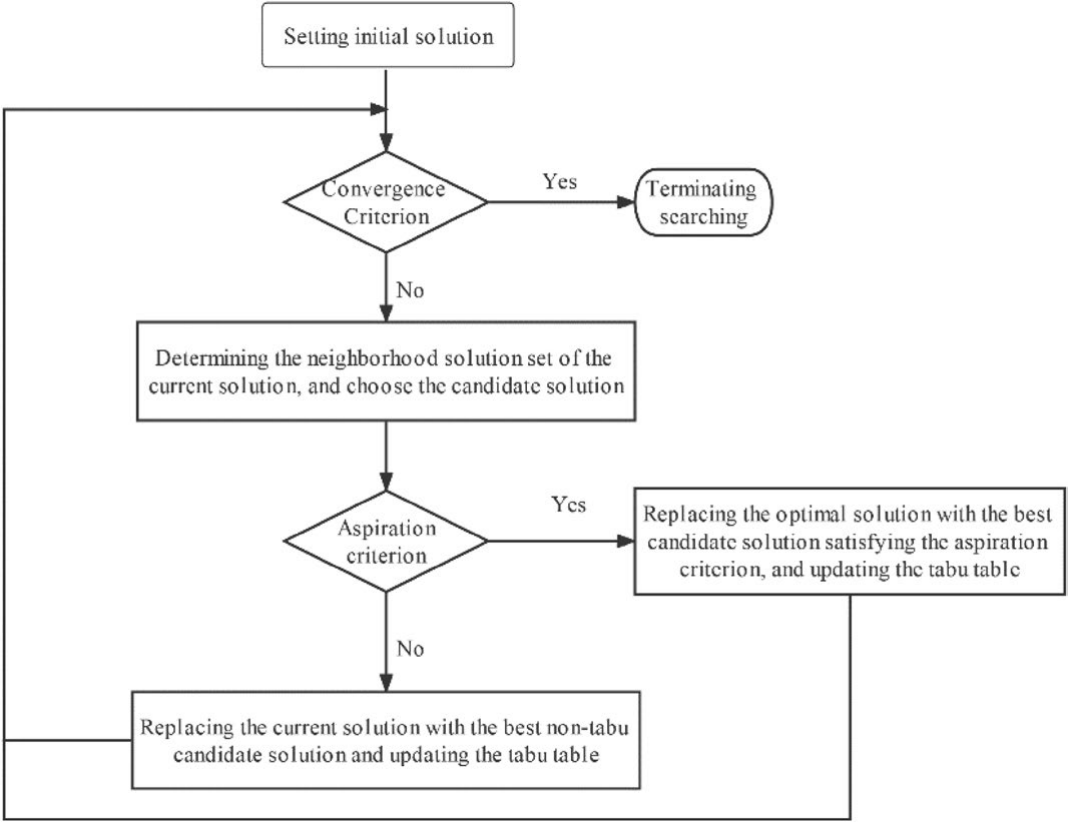
Detailed steps of the Tabu-algorithm.

From April 2019 to November 2019, a screening program for CKD was conducted in ten rural regions in Shanxi province, namely, Ningwu county, Yu county, Yangqu county, Lin county, Shouyang county, Zezhou county, Huozhou city, Hejin city, Linyi county, Ruicheng county. In total, 13, 550 villagers participated in the program, and 12,269 were finally enrolled in this study with 5198 men and 7071 women. Informed consent was signed by all study participants and this study was approved by the Ethics Committee of Shanxi Provincial hospital, with reference number 2021213. Also, All experiments and methods were performed in accordance with the relevant guidelines and regulations. Inclusion criteria included residents over 40 years old. Exclusion criteria included: (1) incomplete recorded data, (2) those less than 40 years old, (3) those unwilling to cooperate, and (4) pregnant women with and history of substance abuse. The flowchart of this study is shown in Fig. 4.

### Data collection

Data were collected by questionnaire, physical examinations, and laboratory analyses. The questionnaire comprises sociodemographic information (sex, age, annual income, education level), medical history (hypertension, diabetes, dyslipidemia, cerebrovascular disease) and lifestyles (exercise, smoking, alcohol consumption, dietary habits). The questionnaire was conducted online or offline and was completed by the subjects themselves or their families. Physical examinations were conducted by medical staff or trained medical students at Shanxi Provincial hospital. They consist of height, weight and blood pressure which were measured two times and then we calculated the mean value. Body Mass Index (BMI) was calculated as weight in kilograms divided by the square of height in meters. Besides, fasting venous blood was taken from participants for high-density lipoprotein cholesterol (HDL-C), low-density lipoprotein cholesterol (LDL-C), total cholesterol (TC), triglyceride (TC), glycosylated hemoglobin (GHb), fasting plasma glucose (FPG), homocysteine (Hcy). Additionally, morning urine was garnered for α1-Microgloblobumin(α1-MG), urine creatinine and microalbuminuria, and then we calculated ACR and MCR.

### Bayesian networks

A Bayesian network consists of a directed acyclic graph whose nodes represent random variables and links express dependencies between nodes^44^. If one link is from variable A to variable B (A → B), it means variable A has an influence on variable B or variable A is the risk factor for variable B. Also, variable A is called the parent node of variable B, and variable B is the child node of variable A. Another important concept in Bayesian networks is that each child node has a conditional probability distribution that measures the effects of its predictor variables (parents). This is given by P (Xi| pa (Xi)), where P is the conditional probability, Xi represents each node and pa (Xi) represents the parents of node Xi^45^. Learning from Bayesian networks includes structural learning and parametric learning. According to the taboo search algorithm, the model is optimized to find the optimal model. Parameter learning: Based on structural learning, the maximum likelihood estimation method is used to calculate the conditional probability of each node of the network.

### Tabu-Search algorithm

Tabu-Search (TS)^30^, proposed by Professor Fred Glover in 1986, is an intelligent global optimization algorithm^21^. The algorithm mainly simulates human memory function and obtains the global optimal solution by searching the local neighbourhood step by step. In order to avoid falling into local optimality and repeated iterations, the tabu search algorithm adopts a tabu table, which can be obscured by the search area. As such, the algorithm could avoid detour search, and records the solution process of mobile search and selection. Meanwhile, the table exempts some good states in the tabu area, so as to ensure the diversity of search and achieve global optimization^46^. The procedures of the algorithm^47^ include the following six ones: (1) setting parameters and the initial solution x. (2) Determine whether the convergence criterion is met? If yes, the optimization result x is output; If not, continue with the next step. (3) Determine the neighborhood of the current solution x and choose the candidate solution. (4) Determine whether the candidate solution of the search meets the contempt criterion? If so, replace x with the best candidate solution y satisfying the aspiration criterion to become the new optimal solution, and update the tabu table with the taboo object corresponding to y, and then move to step two; If not, continue with the next step. (5) replacing the current optimal solution with the best state corresponding to the non-taboo object in the candidate solution set, and update the tabu table with the corresponding taboo object, and then switching to step two. (6) Repeating this process until the convergence criterion^48^ is met, and finish the searching (Fig. 4).

### Definitions

Annual income/ educational levels, smoking/alcohol consumption status, previous medical history and lifestyle were obtained from questionnaire. The categories of annual income were divided into 4 parts, namely, < 5000 Yuan, 5000–10,000 Yuan, 10,000–20,000 Yuan, and > 20,000 Yuan. The categories of education level consisted of ≤ Primary school, ≤ Middle school, ≤ High school, ≥ college. Smoking was divided into Yes or No. Alcohol consumption was defined as Always (over 100 g/time and 3 times/week), Sometimes (< 3 times/week or less than 100 g/time) and Seldom. Exercise was classified into Never or Always (≥ 3 times/weeks and ≥ 30 min/time with intensity over moderate walking). Salt consumption was defined as Light, Balanced, and Salt. Diet was defined as vegetable, balanced and meat. Previous medical history was defined as Yes or No.

Under the Chinese Guidelines on Prevention and Treatment of Dyslipidemia in Adults published in 2007^49^, TC ≥ 6.22 mmol/L was defined as hypercholesterolemia; TG ≥ 2.26 mmol/L was defined as hypertriglyceridemia; LDL-C ≥ 4.14 mmol/L was defined as high levels of low-density lipoprotein cholesterol; HDL-C < 1.04 mmol/L was defined as low levels of high-density lipoprotein cholesterol. HHcy was defined as Hcy > 15 μmol/L^50^. FPG was defined as Normal (< 6.1 mmol/L ), Impaired Fasting Glucose(IFG, 6.1 mmol/L ~ 7.0 mmol/L) and Hyperglycosemia (> 7.0 mmol/L).

Following the Guidelines for the Prevention and Treatment of Type 2 Diabetes in China published in 2021, GHb was defined as Normal (< 6.5 mmol/L) and Abnormal (≥ 6.5 mmol/L). SBP was defined as High(≥ 140 mmHg), Normal(120 ~ 140 mmHg) and low(< 120 mmHg), and DBP was defined as High(≥ 90 mmHg), Normal(80 ~ 90 mmHg) and Low(< 80 mmHg)^51^. As per the standards established for Chinese by the Department of Disease Control, Ministry of Health^52^, BMI classification comprised underweight(< 18.5 kg/m^2^), normal(18.5 ~ 24.0 kg/m^2^), overweight(24.0 ~ 28.0 kg/ m^2^), obesity (≥ 28 kg/m^2^). ACR equals to mAlb/Ucr*8.84; MCR equals α-1 MG/Ucr*8.84. ACR ≥ 30 mg/g was defined as increased ACR and MCR > 23 mg/g was defined as increased MCR.

### Statistical methods

Statistical descriptions are expressed as percentages (%), and comparisons were tested using chi-square tests. The chi-square test for statistically significant variables was incorporated into Logistic regression analysis. Statistically significant variables detected in Logistic regression were incorporated into Bayesian network modeling. The structure learning of BNs is carried out by using the “tabu” function in the package “bnlearn” in R 4.2.0 software (R Development Core Team). We also compared its performance with Hill-climbing algorithm, which was achieved using “hc” function. Besides, the parameter learning of Bayesian networks is carried out using Maximum Likelihood Estimate, and the Bayesian network and conditional probability distribution table are plotted by Netica software (Norsys Sofware Corp., Vancouver, BC, Canada).

## Supplementary Information

Supplementary Figure 1.

Supplementary Figure 2.

Supplementary Figure 3.

## Data Availability

The data that support the findings of this study are available from the corresponding author upon reasonable request.
