# Supplementary material for: Using Bayesian networks with Tabu-search algorithm to explore risk factors for hyperhomocysteinemia
Source: Sci Rep. 2023 Jan 28;13:1610. doi: 10.1038/s41598-023-28123-z (PMC9884210; doi:10.1038/s41598-023-28123-z)
Supplement: Supplementary file 1 — Supplementary Figure 1. [file 41598_2023_28123_MOESM1_ESM.pdf]

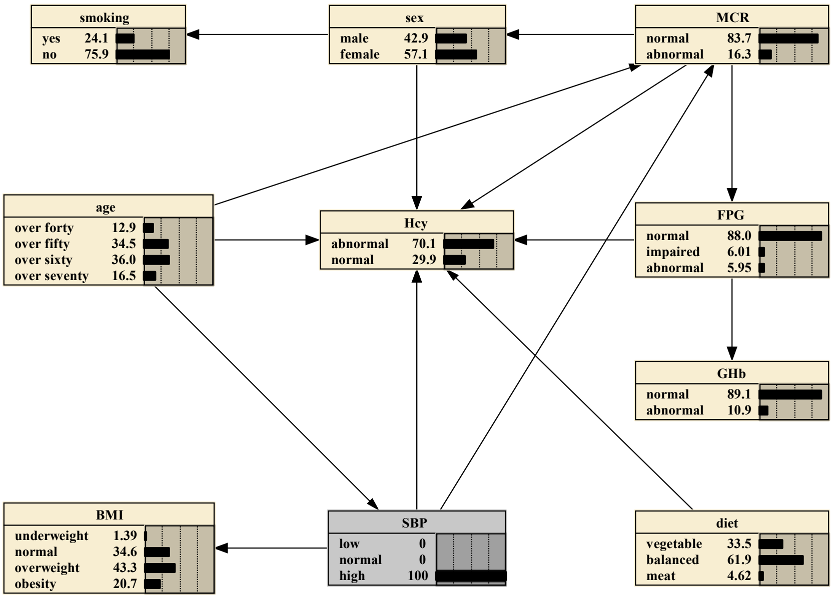

Supplementary Figure 1 Bayesian reasoning for HHcy under high SBP. The figure was plotted using Netica (www.norsys.com).

Node represents variable, and directed edges represent probabilistic dependence between connected nodes. If one is subject to high SBP, the probability increases from the prior probability to P (HHcy|high SBP)=0.701.
