# Supplementary material for: Using Bayesian networks with Tabu-search algorithm to explore risk factors for hyperhomocysteinemia
Source: Sci Rep. 2023 Jan 28;13:1610. doi: 10.1038/s41598-023-28123-z (PMC9884210; doi:10.1038/s41598-023-28123-z)
Supplement: Supplementary file 2 — Supplementary Figure 2. [file 41598_2023_28123_MOESM2_ESM.pdf]

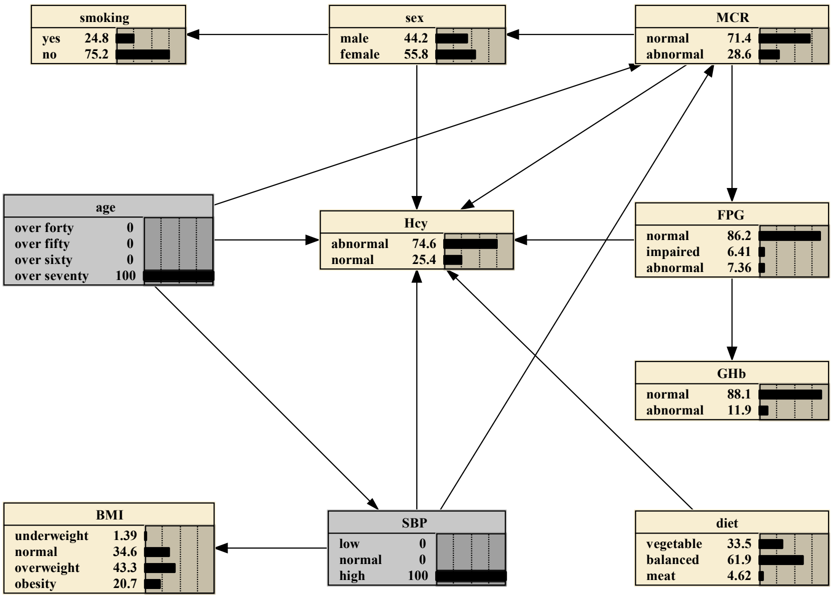

Supplementary Figure 2 Bayesian reasoning for HHcy under high SBP and with an age over seventy. The figure was plotted using Netica (www.norsys.com).

Node represents variable, and directed edges represent probabilistic dependence between connected nodes. If the person' s age is over seventy, the probability rises to P (HHcy|high SBP, 71-91 years)=0.746.
