# Supplementary material for: Using Bayesian networks with Tabu-search algorithm to explore risk factors for hyperhomocysteinemia
Source: Sci Rep. 2023 Jan 28;13:1610. doi: 10.1038/s41598-023-28123-z (PMC9884210; doi:10.1038/s41598-023-28123-z)
Supplement: Supplementary file 3 — Supplementary Figure 3. [file 41598_2023_28123_MOESM3_ESM.pdf]

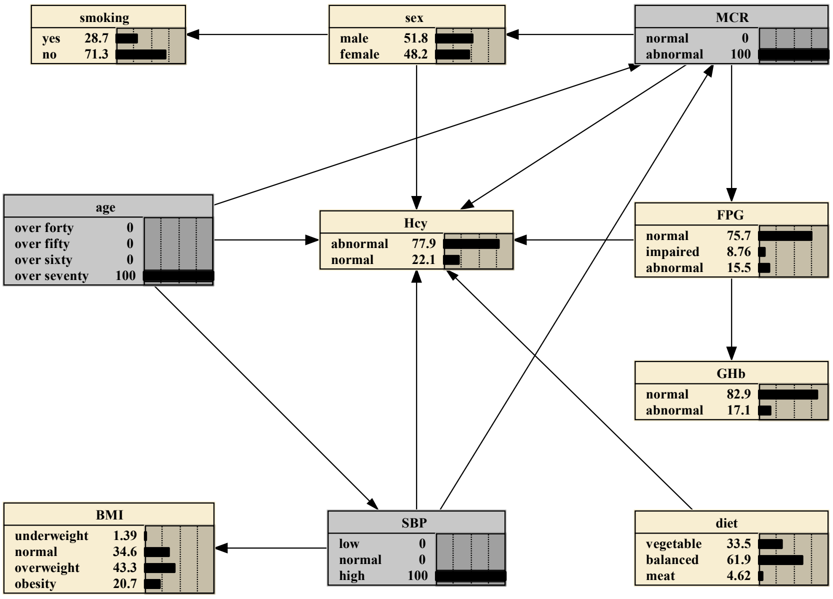


Supplementary Figure 3 Bayesian reasoning for HHcy under high SBP and with an age over seventy and with abnormal MCR . The figure was plotted using Netica (www.norsys.com).

Node represents variable, and directed edges represent probabilistic dependence between connected nodes. If the person has abnormal MCR, the probability rises to P (HHcy|high SBP, 71-91 years, abnormal MCR)=0.779.
